# Intergeneration transfer of diet patterns? Parental self-report of diet and their report of their young adult children with ASD

**DOI:** 10.1371/journal.pone.0263445

**Published:** 2022-02-08

**Authors:** Sibylle Kranz, Julia Lukacs, Jason Bishop, Martin E. Block

**Affiliations:** 1 Department of Kinesiology, University of Virginia, Charlottesville, Virginia, United States of America; 2 Department of Physical Education, Auburn University Montgomery, Montgomery, Alabama, United States of America; East Stroudsburg University, UNITED STATES

## Abstract

Autism Spectrum Disorder (ASD) affects two percent of American children and often results in neophobia, hypersensitivity to foods, and firmly set food preferences, leading to higher proportions of individuals suffering from diet-related chronic diseases. Our objective was to conduct an explorative pilot study to examine parents’ perception of food intake for themselves and their young adult children with ASD. We employed comparative analysis to discover potential pathways to improve diet quality and lower the risk for chronic disease in individuals with ASD. Data from an online survey in n = 493 parent-child dyads on parentally reported intake patterns, food group, and food consumption was analyzed using kappa statistics to determine the level of agreement between reported parental and child intake patterns, body weight status and activity level. Average age was 48 years for parents and 22 years for their children, respectively. Parent-child agreement for obesity was high. We found very strong agreement between the reported diet variety (kappa = 0.82) and changing daily intake (kappa = 0.63) and strong agreement for some vegetable intake patterns (kappa = 0.61 for orange, white, and starchy vegetables) but not in meat intake (no agreement). Results of this study indicate evidence for perceived intergenerational transfer of dietary intake patterns, which may offer effective approaches to change parental diet, to subsequently improve diet quality in young adults with ASD and prevent diet-related chronic diseases in individuals with ASD.

## Introduction

On average, Autism Spectrum Disorder (ASD) affects two percent of American children [1]. The disorder, characterized for its impact on social development, also affects eating habits and nutrition, including hypersensitivity to foods, neophobia (the fear of trying new foods), and firmly set preferences for some but complete refusal of other tastes or textures of food [2, 3]. Up to 90% of autistic children present maladaptive eating habits of varying levels, which include severe food selectivity (children with ASD commonly prefer foods that do not have strong odor, taste, smell and coarse texture), rapid eating, and food rejection- all of which influence specific food preferences [4–7]. Such habits contribute to lower consumption of nutrient-dense foods and lower average dietary intakes of protein, animal fat, calcium, and phosphorus in children with ASD compared to non-autistic children [8, 9]. ASD is also associated with lower diet variety, contributing to health risks such as obesity and vitamin deficiencies- namely calcium, iron, zinc, vitamin A, vitamin and D [3, 10, 11]. Diet variety is well known as strong indicator for better diet quality, since individuals with high diet variety likely meet nutrient and food-group requirements and are at lower risk for diet-related chronic diseases [12].

Individuals with ASD commonly exhibit gastrointestinal maladies including insufficiencies in digestive enzymes, diet-obtained protein antibodies, and compromised gut barriers [13, 14], which may be related to lack of a habitually healthy diet, which is integral to maintaining good health and preventing chronic disease [15, 16]. Due to the dietary preferences associated with the disease, individuals with ASD are disproportionately at larger risk for diet-related chronic diseases, such as obesity, Type 2 diabetes, and cardiovascular disease [17].

Dietary intake is a result of learned behavior, such as food intake patterns and food preferences, which are formed at a very early age and are largely influenced by parents or primary caregivers, who serve as models for food-related behaviors [18]. To date, there is very little information on the possible influence of parental dietary patterns on the diet patterns of their child with ASD. The aim of this pilot study was to close this gap by exploring the potential for perceived intergenerational transfer of dietary intake patterns between parents and their adult child with ASD. Based on data provided by Park et al. and Panossian et al., increased severity of ASD has been found to be associated with problematic dietary practices [7], and lower food variety [10], therefore, we also conducted stratified analysis by parental and child weight status and the child’s level of ASD and to assess if there was agreement of higher levels of ASD with lower diet variety. We present data from an original research project, our data has not been published elsewhere, we conducted kappa statistics to assess level of agreement between the parent’s responses for themselves compared to the response for their child, the statistical approach is described in detail in the Statistical Analysis section, our conclusions are discussed in the Discussion section and based on the results presented in the tables and Result section, all data points are available in the tables, and our research project was approved by the Institutional Research Board (IRB).

## Materials and methods

This study was approved by the local Institutional Review Board for Social and Behavioral Human Subjects Research (IRB# 2018-0462-00. Data was collected through internet-based completion of a survey instrument using parent’s self-reported responses in a *Qualtrics survey* (https://www.qualtrics.com) available between June and July 2018. The survey consisted of 107 questions, including questions about the household’s socio-economic factors, parental age and sex, 28 questions on dietary patterns, and 41 questions about physical activity (reported elsewhere). The dietary questions were developed to obtain information on a number of factors of intake patterns as well as the consumption of commonly under-consumed foods in individuals with ASD. Diet variety was defined as consuming different food groups and several of the options within each of the food group (vegetables, meats, grains, and oils/fats). Several of the questions were exploratory, to provide data to currently unknown factors, such as the individuals preference temperature of beverages or if sweet or salty snacks are preferred. Recruitment of participants was facilitated through a national “Autism Speaks’” database, which included schools and other facilities with programs serving individuals with ASD of all ages. In addition, an internet search was conducted to identify schools/facilities in 30 of the largest states (by population) in the United States that served young adults with ASD. Directors or similar school administrators from these schools/facilities were contacted via email to explain the purpose of the study and to ask them to distribute an email to parents of young adults with ASD to invite participation. Directors were also asked to display study flyers in a high traffic and high visibility area in their facility. A $75 donation or Visa gift card was offered to the schools for their help with recruitment and parents were offered a $20 Amazon gift card for completing the survey. Interested parents of children meeting the inclusion criteria consented to the study by navigating to the study URL and choosing “agree” to the consent statement. Parents were subsequently directed to the full survey. The inclusion criteria for the study were: 1) participant was the parent (or primary caregiver) of child with ASD that is between the ages of 18 and 28, 2) the child’s ASD diagnosis was from a physician or school psychologist, 3) the child did not have restrictions from a physician regarding exercise, and 4) the child resided and was under care in same residence as the parent (or primary caregiver).

**Fig 1:**
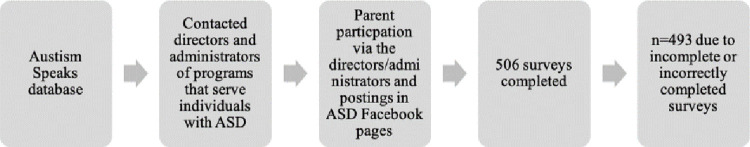
Recruitment of subjects.

After multiple attempts at contacting the schools fewer than 20 directors of the targeted facilities responded and calls for participation in this study were place on several ASD Facebook pages, and the incentive for parents to participate was increased to $40. This change resulted in slightly over 500 parents completing the survey. Analysis of the surveys showed that several of the surveys were incomplete or were completed in extremely short time, indicating inaccurate responses. The research team eliminated surveys that were incomplete or completed within a non-feasible timeframe (surveys which were completed in less than four minutes), resulting in a total sample size of 493 parent-child dyads (Fig 1), of which n = 77 did not report their child’s sex and n = 275 parents reported having a male child and n = 141 parents had a female child with ASD. Not all parents answered all questions, leading to varying number of responses considered in our analysis.

The survey for this study was designed to obtain information from the parent on his/her own diet and his/her adult child with ASD; the questionnaire had 14 questions regarding dietary intake of the parent and the same 14 questions on the parental report of the intake habits of the child. Twenty-two questions were for demographical information for the parent-child dyad. Prior to launch, this survey was reviewed for validity by experts in the area of nutrition and physical activity in individuals with ASD. Furthermore, the survey was piloted in a small sample of parents with children with ASD in the local geographical area (n = 5). In order to examine the effect of body weight status, weight categories were established using the CDC guidelines for Body Mass Index for adults (in participants 19 years and older) and children [19].

### Statistical analysis

Survey results are presented in percent of responses separately for the parent’s self-reported diet and the parental report for their adult child with ASD. The appropriate statistical procedure to assess levels of agreement between the reported responses is the kappa statistic [20]. In the medical field, the kappa statistic is commonly used for comparison of rater reliability and comparison of accurate and measured values [20]. This method allows to assess data agreement by chance or “expected” [21]. Since our data relies on the report of the parent’s own dietary habits and the parent-report of their child’s dietary habits, we elected to use the kappa statistic to measure the level of agreement between responses to explore the level of perceived similarity. We used the statistical program SPSS (IBM), populated tables with the responses for parental his-/herself and the child to then calculate the kappa values, followed by chi-square tests comparing the proportions in the table to ascertain the corresponding p-value for the comparison. Thus, we assessed a) the level of agreement of the parents’ response for themselves and for their child and b) the level of significance of the observed data being statistically different (p<0.05 was deemed significant). The following classifications for kappa value for agreement were used: very strong (kappa = 0.81–1), substantial (kappa = 0.61–0.8), moderate (kappa = 0.41–0.6), fair (kappa = 0.21–0.4), and poor (0.01–0.2). Statistically significant agreement between the responses was set at kappa value>0.61(20), and p-values of p<0.001, p<0.05, and p<0.1 were noted. The tables reflect the responses to the survey questions as percent and the level of agreement between the parent’s responses overall (Table 2), responses and agreement stratified by parent or child body weight status independently (Table 3), and by child’s ASD level (Table 4).

## Results

A total of 493 parents (34% males and 41% females) completed the survey (n = 275 parents reported having a male child and n = 141 parents had a female child with AS, and n = 77 did not report their child’s sex), providing information on their and their adult child demographic characteristics and dietary intake patterns. Parents reported family sizes ranging from two to seven members, 85% of parents were married, 85% owned their house, and 30% had a yearly income of $100,000 or less (complete description of the sample can be found elsewhere, in review). Table 1 reflects the sample characteristics and the level of agreement between parent and child weight status. Approximately 25% of the sample did not indicate the parent’s or child’s sex or height and weight. The average age of the parents was 48 years and 22 years for their children. Only 9.9% of the young adult children with ASD and 8.2% of the parents were classified as obese (kappa = 0.61). The classifications of “underweight” and “overweight” body weight status had significant fair agreement between the parent-child dyads, “healthy weight” had moderate, and “obese” had substantial agreement between parents and their associated adult child with ASD.

**Table 1:** Demographics (N = 488).

Demographic Variable	Child	Parent	Parent-Child Agreement
Sex	%	%	-
Female	25.3	34.2	-
Male	49.3	40.5	-
Not specified	0.2	0	-
Missing	25.3	25.3	-
BMI			Kappa and *P*-value
Underweight	9.1	7.2	0.29 *p<0*.*001*
Healthy	19.0	21.7	0.45 *p<0*.*001*
Overweight	12.0	12.5	0.28 *p<0*.*001*
Obese	9.9	8.2	0.61^2^ *p<0*.*001*
Missing	50.0	50.4	-
Mean Age	21.76 +/- 3.679	47.61 +/- 6.608	-
Mean BMI	24.24 +/- 9.939	25.09 +/- 25.38	-
ASD Level			
ASD Level 1	47.6	-	-
ASD Level 2	21.7	-	-
ASD Level 3	7.9	-	-
ASD Level 4	3.5	-	-
Missing	19.4	-	-

ASD = Autism Spectrum Disorder

Parental responses to questions on food intake patterns and food group consumption or preference are shown in Table 2. Our data indicate that parents perceive their diet variety and their children’s diet variety to be similar, in that 81.6% of parents reported that they consumed a variety of foods and 76.8% of parents reported that their child’s diets also reflected consumption of a variety of food groups (kappa = 0.82). Furthermore, 76% of the responses pertaining to the parents and 66.8% of responses pertaining to the children indicated they did not consume the same foods every day (substantial agreement, kappa = 0.63). The data indicated fair agreement for overall snacking (kappa = 0.36) and snacking on sweet and soft foods or fruits/vegetables (kappa = 0.22 and kappa = 0.26, respectively). Beverage consumption had fair agreement for drinking at least eight glasses of water per day (kappa = 0.54), preference for drinking hot beverages (kappa = 0.25), cold beverages (kappa = 0.16), or both (hot or cold beverages) was fair (kappa = 0.31). About 50% or parents reported that they and their children were taking nutrition supplements (substantial agreement, kappa = 0.61), but there was only fair agreement among the types of supplements taken (absolute value of kappa range = 0.21–0.39). Parents reported that approximately 33% of children and 52% of parents were “picky eater”, which had moderate agreement (kappa = 0.52). The question “Do you now like some of the foods you didn’t like as a child?” was answered with “yes” by 64% of the parents and had moderate agreement (kappa = 0.48) with their response for their child. In the investigation of food group intakes, we found the highest levels of agreement in the consumption of orange, white and starchy vegetables (kappa = 0.61), margarine (kappa = 0.70), and olive oil/vegetable oils (kappa = 0.68) and moderate agreement with dark leafy greens (kappa = 0.45), cruciferous (kappa = 0.57), onion family varieties (kappa = 0.51), and eating a combination of vegetable groups (kappa = 0.59). Likewise, grain consumption had moderate agreement (white bread (kappa = 0.56), whole grain bread (kappa = 0.51), breakfast cereals (kappa = 0.52), rice (kappa = 0.51), and not eating grains at least five times per week (kappa = 0.51). Oil and fats consumption was similar for margarine (kappa = 0.70), olive oil/vegetable oil or shortening and butter (kappa = 0.68), and butter (kappa = 0.60). The only food group for which little agreement was found was the meat group, where only fair agreement was found for the item “cold cuts and other ready-to-eat meats” (kappa = 0.22) and “I eat a combination of these meats at least five times per week” (kappa = 0.23).

**Table 2:** Percent of parent and child response and parent-child agreement to the survey questions (N = 488).

	Parent	Child	Agreement
**1. Do you eat a variety of food groups (different fruits, vegetables, meats, grains, fats and oils) in your daily diet?**			
Response	%	%	Kappa and *P*-value
Yes	81.6	76.8	0.82^1^ p<0.001
No	3.7	8.8	0.40 p<0.001
Missing	14.8	14.3	-
**2. Do you eat the same foods every day?**			
Response	%	%	Kappa and *P*-value
Yes	9.2	18.9	0.32 p<0.001
No	76	66.8	0.63^2^ p<0.001
Missing	14.8	14.3	-
**3. Do you like to snack (eat small amounts between meals)?**			
Response	%	%	Kappa and *P*-value
Yes	68.2	45.5	0.36 p<0.001
No	16.2	0.82	0.22 p<0.001
Sometimes	0	30.3	0
Rarely	0	9.2	0
Missing	15.6	14.1	-
**4. If you snack, which type of snack food do you prefer most of the time?**			
Response	%	%	Kappa and *P*-value
Sweet and Soft	19.1	29.9	0.22 p<0.001
Sweet and Crunchy	10.2	14.5	0.12 p=0.007
Salty and Soft	10.5	5.5	-0.02 *p* = 0.669
Salty and Crunchy	15.6	20.7	0.07 *p* = 0.98
Fruit or Vegetables	18.2	7.4	0.264 p<0.001
No preference	12.2	10.2	0.425 p<0.001
Missing	19.3	16.2	-
**5. Do you drink at least 8 glasses of water every day?**			
Response	%	%	Kappa and *P*-value
Yes	59.8	49	0.540 p<0.001
No	25	36.1	0.443 p<0.001
Missing	15.2	15	-
**6. Which types of beverages/drinks do you prefer?**			
Response	%	%	Kappa and *P*-value
Hot	27.7	12.9	0.250 p<0.001
Cold	18.4	39.8	0.161 p<0.001
Both	38.9	29.9	0.312 p<0.001
Neither	0	3.1	0
Missing	15	14.3	-
**7. Do you take nutrition supplements?**			
Response	%	%	Kappa and *P*-value
Yes	53.1	50.4	0.612^2^ p<0.001
No	32	34.4	0.534 p<0.001
Missing	15	15.2	-
**8. If you take supplements, which type do you take?**			
Response	%	%	Kappa and *P*-value
Daily multivitamin and minerals combined	33.4	38.9	0.389 p<0.001
Single nutrient or minerals	18.2	13.3	0.211 p<0.001
Both	13.7	13.3	0.393 p<0.001
Missing	34.6	34.4	-
**9. Do you remember what type of eater you were when you were less than 5 years old?**			
Response	%	%	Kappa and *P*-value
I do not remember	25.8	9.2	0.243 p<0.001
I ate everything my parents gave me	26	24.2	0.483 p<0.001
I was a picky eater and didn’t like certain foods	33.4	52	0.516 p<0.001
Missing	14.8	14.5	-
**10. Do you now like some of the foods you didn’t like as a child?**			
Response	%	%	Kappa and *P*-value
Yes	63.5	48.2	0.476 p<0.001
No	21.9	35.9	0.362 p<0.001
Missing	14.5	16	-
**11. If you eat different vegetables, which do you eat at least 5 times per week?** *			
Response	%	%	Kappa and *P*-value
Dark leafy greens	13.7	10.6	0.448 p<0.001
Orange	20.5	21.7	0.610^2^ p<0.001
Cruciferous	13.8	13.4	0.568 p<0.001
White and starchy	18.7	22.1	0.607^2^ p<0.001
Onion family	13.4	12.2	0.509 p<0.001
I do not eat any vegetable groups at least 5 times per week	3.9	4.3	0.362 p<0.001
I eat a combination of these vegetable groups at least 5 times per week	9.3	8.2	0.587 p<0.001
Missing	6.7	7.5	-
**12. If you eat different meats, which do you eat at least 5 times per week?** *			
Response	%	%	Kappa and *P*-value
Fish	17.2	12.7	0.117 p<0.001
Chicken or Turkey	24.7	27.2	0.194 p<0.001
Red meat	26.4	29.4	0.198 p<0.001
Cold cuts and other ready-to-eat meats	11.2	12.8	0.216 p<0.001
I do not eat meats at least 5 times per week	3.7	3	0.121 p<0.001
I eat a combination of these meats at least 5 times per week	9	7	0.229 p<0.001
Missing	7.8	7.9	-
**13. If you eat different grains, which do you eat at least 5 times per week?** *			
Response	%	%	Kappa and *P*-value
White bread	19	24.6	0.555 p<0.001
Whole grain breads	21.5	21.8	0.513 p<0.001
Breakfast Cereals	18	21.4	0.524 p<0.001
Rice	10.2	10.5	0.509 p<0.001
Pasta	11.9	1.2	0.383 p<0.001
I do not eat grains at least 5 times per week	2.2	1.9	0.508 p<0.001
I eat a combination of these grains at least 5 times per week	2.2	7.3	0.270 p<0.001
Missing	9.7	8.2	-
**14. If you use different oils and fats, which do you eat at least 5 times per week?** *			
Response	%	%	Kappa and *P*-value
Butter	24.1	24.8	0.598 p<0.001
Margarine	19.1	18.8	0.701^2^ p<0.001
Olive oil or other vegetable oil/shortening or lard	31.9	31.1	0.682^2^ p<0.001
I do not eat oils and fats at least 5 times per week	4.7	6.4	0.370 p<0.001
I eat a combination of these oils and fats at least 5 times per week	10.4	9.7	0.559 p<0.001
Missing	9.7	9.3	-

^1^indicates a very strong Kappa value

^2^indicates a substantial Kappa value

*indicates question was not mutually exclusive

Turquoise: indicates p<0.001

Bright Green: indicates p<0.05

Yellow: indicates p<0.1

Most notable dietary habits of young adult children with ASD were that many children (45.5%) ate snacks, with sweet and soft snacks reported as the most popular type of snack (preference of 29.9% children). Parental reports showed that 49% of children drink at least eight glasses of water a day, with the highest preference for cold drinks (39.8%). Supplement use, most commonly a combined daily multivitamin and mineral (38.9%), was reported for 50.4% of children and 52% of young adult children with ASD were reportedly picky eaters, however 48.2% of the children now eat foods that they previously did not eat.

A high percentage of parents reported having a varied diet (76.8%), meaning they reportedly consume several of the types of vegetables, meats, grains, and oil/fats. Also, 68.2% of parents reported snacking, with the most preferred snack choices being sweet and soft (19.1%) and fruits or vegetables (18.2%). Parents reported drinking at least eight glasses of water per day (59.8%) and parents preferred both hot and cold beverages (38.9%). Similar to the children, parents reported supplement use (53.1%), and the most preferred supplement of choice was a daily multivitamin and mineral (33.4%). When asked about previous eating behaviors, 33.4% of parents reported being picky eaters, but a large percentage reported eating foods now that they previously did not eat (63.5%).

Results reflected in Table 3 show reported responses by parental and child BMI status. Results showed that similar to other researchers, we also found no consistent significant agreement between parental or child BMI classification and the responses to the questions. There were some significant agreements between responses and BMI status, however, kappa values did not show high levels of agreement, indicating a statistical significance despite low level of agreement. For example, our data showed significant p-values for parents eating a variety of vegetables (kappa = 0.103, p-value 0.001).

**Table 3:** Percent of reponses and parent-child agreement to the survey questions by parental and child BMI classification (N = 488).

	Parent BMI of Underweight and Healthy	Parent BMI of Overweight	Parent BMI of Obese	Child BMI of Underweight and Healthy	Child BMI of Overweight	Child BMI of Obese
**1. Do you eat a variety of food groups (different fruits, vegetables, meats, grains, fats and oils) in your daily diet?**						
Response	Kappa and *P*-value	Kappa and *P*-value	Kappa and *P*-value	Kappa and *P*-value	Kappa and *P*-value	Kappa and *P*-value
Yes	0.15 p<0.001	0.08 p<0.001	0.02 *p* = 0.16	0.153 p<0.001	0.03 p=0.066	0.03 p=0.073
No	0.01 *p* = 0.702	-0.02 *p* = 0.318	0.09 p<0.001	0.035 p=0.024	0.035 p=0.094	0.06 p=0.006
Missing	-	-	-	-	-	-
**2. Do you eat the same foods every day?**						
Response	Kappa and *P*-value	Kappa and *P*-value	Kappa and *P*-value	Kappa and *P*-value	Kappa and *P*-value	Kappa and *P*-value
Yes	0.02 *p* = 0.66	0.06 *p* = 0.177	0.15 p=0.001	0.296 p<0.001	-0.06 *p* = 0.167	0.13 p=0.003
No	0.06 p<0.001	0.03 p=0.004	0.01 *p* = 0.269	0.005 *p* = 0.707	0.04 p<0.001	0.01 *p* = 0.387
Missing	-	-	-	-	-	-
**3. Do you like to snack (eat small amounts between meals)?**						
Response	Kappa and *P*-value	Kappa and *P*-value	Kappa and *P*-value	Kappa and *P*-value	Kappa and *P*-value	Kappa and *P*-value
Yes	0.22 p<0.001	0.12 p<0.001	0.07 p=0.001	0.100 p=0.017	-0.00 *p* = 0.89	0.05 *p* = 0.142
No	-0.04 p=0.047	-0.05 p=0.018	-0.01 *p* = 0.675	0.038 p=0.036	0.02 *p* = 0.143	0.02 *p* = 0.199
Sometimes	0	0	0	0.043 *p* = 0.007	0.01 p=0.009	0.01 *p* = 0.558
Rarely	0	0	0	-0.001 *p* = 0.854	0.01 *p* = 0.273	0.01 *p* = 0.356
Missing	-	-	-	-	-	-
**4. If you snack, which type of snack food do you prefer most of the time?**						
Response	Kappa and *P*-value	Kappa and *P*-value	Kappa and *P*-value	Kappa and *P*-value	Kappa and *P*-value	Kappa and *P*-value
Sweet and Soft	0.01 *p* = 0.854	-0.01 *p* = 0.868	-0.02 *p* = 0.625	0.004 *p* = 0.931	-0.03 *p* = 0.408	-0.08 p=0.035
Sweet and Crunchy	0.10 p=0.002	-0.01 *p* = 0.754	-0.03 *p* = 0.206	0.036 p=0.041	0.05 p=0.016	0.00 *p* = 0.879
Salty and Soft	0.05 p=0.007	0.05 p=0.026	-0.01 *p* = 0.794	0.024 p=0.073	0.04 p=0.086	0.01 *p* = 0.484
Salty and Crunchy	0.03 p=0.094	0.04 p=0.081	0.02 *p* = 0.316	0.101 p<0.001	-0. *p* = 0.907	0.00 *p* = 0.959
Fruit or Vegetables	0.019 *p* = 0.32	0.041 p=0.039	-0.030 *p* = 0.113	0.014 *p* = 0.343	-0.004 *p* = 0.85	0.001 *p* = 0.942
No preference	0 *p* = 0.999	-0.015 *p* = 0.454	0.148 p<0.001	-0.035 p=0.017	0.035 p=0.093	0.175 p<0.001
Missing	-	-	-	-	-	-
**5. Do you drink at least 8 glasses of water every day?**						
Response	Kappa and *P*-value	Kappa and *P*-value	Kappa and *P*-value	Kappa and *P*-value	Kappa and *P*-value	Kappa and *P*-value
Yes	0.101 p=0.004	0.125 p<0.001	0.064 p=0.004	0.010 *p* = 0.806	0.123 p<0.001	0.044 *p* = 0.132
No	0.049 p=0.009	-0.025 *p* = 0.183	0.001 *p* = 0.934	0.100 p<0.001	-0.030 p=0.047	0.024 *p* = 0.129
Missing	-	-	-	-	-	-
**6. Which types of beverages/drinks do you prefer?**						
Response	Kappa and *P*-value	Kappa and *P*-value	Kappa and *P*-value	Kappa and *P*-value	Kappa and *P*-value	Kappa and *P*-value
Hot	0.109 p=0.013	0.069 p=0.086	0.039 *p* = 0.278	-0.027 *p* = 0.482	-0.016 *p* = 0.709	-0.034 *p* = 0.442
Cold	-0.19 *p* = 0.329	0.033 p=0.095	-0.46 p=0.015	0.112 p<0.001	0.014 *p* = 0.327	0.057 p<0.001
Both	0.068 p<0.001	0.006 *p* = 0.694	0.056 p<0.001	-0.014 *p* = 0.440	0.003 *p* = 0.853	0.007 *p* = 0.681
Neither	0	0	0	0.022 p=0.037	0.091 p<0.001	-0.023 *p* = 0.173
Missing	-	-	-	-	-	-
**7. Do you take nutrition supplements?**						
Response	Kappa and *P*-value	Kappa and *P*-value	Kappa and *P*-value	Kappa and *P*-value	Kappa and *P*-value	Kappa and *P*-value
Yes	0.238 p<0.001	0.086 p=0.004	-0.007 *p* = 0.789	0.065 *p* = 0.112	0.027 *p* = 0.311	-0.044 *p* = 0.123
No	-0.022 *p* = 0.224	0.010 *p* = 0.567	0.054 p=0.001	0.077 p<0.001	0.028 p=0.074	0.082 p<0.001
Missing	-	-	-	-	-	-
**8. If you take supplements, which type do you take?**						
Response	Kappa and *P*-value	Kappa and *P*-value	Kappa and *P*-value	Kappa and *P*-value	Kappa and *P*-value	Kappa and *P*-value
Daily multivitamin and minerals combined	0.009 *p* = 0.843	0.022 *p* = 0.561	-0.015 *p* = 0.643	0.014 *p* = 0.754	-0.064 p=0.041	-0.063 p=0.059
Single nutrient or minerals	0.097 p<0.001	-0.013 *p* = 0.504	0.001 *p* = 0.954	0.076 p<0.001	0.028 *p* = 0.167	0.018 *p* = 0.389
Both	0.034 p=0.069	0.039 p=0.055	-0.018 *p* = 0.377	0.036 p=0.04	0.009 *p* = 0.643	-0.035 p=0.088
Missing	-	-	-	-	-	-
**9. Do you remember what type of eater you were when you were less than 5 years old?**						
Response	Kappa and *P*-value	Kappa and *P*-value	Kappa and *P*-value	Kappa and *P*-value	Kappa and *P*-value	Kappa and *P*-value
I do not remember	0.218 p<0.001	-0.048 *p* = 0.239	-0.015 *p* = 0.685	0.186 p<0.001	0.020 *p* = 0.645	-0.062 *p* = 0.153
I ate everything my parents gave me	0.057 p=0.003	0.116 p<0.001	-0.007 *p* = 0.663	0.053 p=0.004	0.059 p=0.001	0.064 p<0.001
I was a picky eater and didn’t like certain foods	-0.033 p=0.073	-0.018 *p* = 0.280	0.06 p<0.001	0.006 *p* = 0.715	-0.005 *p* = 0.671	0.013 *p* = 0.295
Missing	-	-	-	-	-	-
**10. Do you now like some of the foods you didn’t like as a child?**						
Response	Kappa and *P*-value	Kappa and *P*-value	Kappa and *P*-value	Kappa and *P*-value	Kappa and *P*-value	Kappa and *P*-value
Yes	0.207 p<0.001	0.083 p=0.001	0.037 p=0.077	-0.069 p=0.097	0.111 p<0.001	0.056 p=0.055
No	-0.023 *p* = 0.235	0.004 *p* = 0.843	0.025 *p* = 0.174	0.134 p<0.001	-0.020 *p* = 0.186	0.010 *p* = 0.512
Missing	-	-	-	-	-	-
**11. If you eat different vegetables, which do you eat at least 5 times per week?** *						
Response	Kappa and *P*-value	Kappa and *P*-value	Kappa and *P*-value	Kappa and *P*-value	Kappa and *P*-value	Kappa and *P*-value
Dark leafy greens	0.171 p<0.001	0.060 p=0.001	-0.019 *p* = 0.258	0.022 *p* = 0.61	0.016 *p* = 0.397	0.004 *p* = 0.827
Orange	0.023 *p* = 0.164	0.067 p=0.049	-0.050 p<0.001	-0.035 p=0.042	-0.019 *p* = 0.539	-0.039 p=0.007
Cruciferous	0.098 p<0.001	0.066 p<0.001	-0.004 *p* = 0.911	0.080 p<0.001	0.029 p=0.099	0.048 *p* = 0.23
White and starchy	0.020 *p*<0.249	0.055 p<0.001	-0.041 p=0.03	0.040 p=0.02	0.007 *p* = 0.597	-0.036 p=0.013
Onion family	0.028 *p* = 0.134	0.053 p=0.004	-0.046 p=0.006	-0.022 *p* = 0.233	-0.014 *p* = 0.448	-0.019 *p* = 0.32
I do not eat any vegetable groups at least 5 times per week	-0.021 *p* = 0.213	0.012 *p* = 0.566	0.039 p=0.071	0.025 *p* = 0.114	0.059 p=0.005	0.058 p=0.006
I eat a combination of these vegetable groups at least 5 times per week	0.017 *p* = 0.384	-0.005 *p* = 0.795	0.103 p<0.001	0.006 *p* = 0.759	0.029 *p* = 0.148	0.066 p=0.001
Missing	-	-	-	-	-	-
**12. If you eat different meats, which do you eat at least 5 times per week?** *						
Response	Kappa and *P*-value	Kappa and *P*-value	Kappa and *P*-value	Kappa and *P*-value	Kappa and *P*-value	Kappa and *P*-value
Fish	0.170 p<0.001	0.025 *p* = 0.149	-0.047 p=0.003	-0.002 *p* = 0.96	0.032 p=0.079	-0.032 p=0.094
Chicken or Turkey	0.080 p=0.047	0.023 *p* = 0.104	-0.041 p=0.001	0.008 *p* = 0.609	0.018 *p* = 0.505	-0. 043 p=0.001
Red meat	0.061 *p* = 0.120	0.018 *p* = 0.197	-0.030 p=0.013	0.031 p=0.041	0.004 *p* = 0.705	-0.047 p=0.085
Cold cuts and other ready-to-eat meats	0.143 p=0.001	0.095 p<0.001	-0.012 *p* = 0.513	0.107 p<0.001	0.059 p=0.001	-0.013 *p* = 0.506
I do not eat meats at least 5 times per week	0.019 *p* = 0.578	0.002 *p* = 0.934	0.050 p=0.02	-0.002 *p* = 0.884	-0.009 *p* = 0.672	0.037 p=0.068
I eat a combination of these meats at least 5 times per week	0.056 *p* = 0.2	0.021 *p* = 0.296	0.099 p<0.001	0.023 *p* = 0.185	-0.001 *p* = 0.947	0.097 p<0.001
Missing	-	-	-	-	-	-
**13. If you eat different grains, which do you eat at least 5 times per week?** *						
Response	Kappa and *P*-value	Kappa and *P*-value	Kappa and *P*-value	Kappa and *P*-value	Kappa and *P*-value	Kappa and *P*-value
White bread	0.076 p=0.079	0.062 p<0.001	-0.026 p=0.075	0.116 p=0.008	0.022 *p* = 0.103	-0.013 *p* = 0.370
Whole grain breads	0.115 p=0.006	0.047 p=0.002	-0.045 p=0.001	0.003 *p* = 0.854	0.087 p=0.007	-0.047 p=0.002
Breakfast Cereals	0.214 p<0.001	0.075 p<0.001	-0.027 p=0.069	0.082 p<0.001	0.051 p=0.001	-0.041 *p* = 0.234
Rice	0.233 p<0.001	0.093 p<0.001	-0.018 *p* = 0.349	0.088 p<0.001	0.039 p=0.045	0.049 p=0.015
Pasta	0.080 p=0.077	0.055 p=0.005	-0.013 *p* = 0.481	0.025 *p* = 0.178	0.040 p=0.026	0.021 *p* = 0.269
I do not eat grains at least 5 times per week	0.035 *p* = 0.227	-0.035 p=0.051	0.014 *p* = 0.48	0.006 *p* = 0.561	-0.025 *p* = 0.181	-0.012 *p* = 0.511
I eat a combination of these grains at least 5 times per week	-0.016 *p* = 0.716	-0.031 *p* = 0.129	0.098 p<0.001	-0.004 *p* = 0.839	-0.022 *p* = 0.275	0.042 p=0.035
Missing	-	-	-	-	-	-
**14. If you use different oils and fats, which do you eat at least 5 times per week?** *						
Response	Kappa and *P*-value	Kappa and *P*-value	Kappa and *P*-value	Kappa and *P*-value	Kappa and *P*-value	Kappa and *P*-value
Butter	0.135 p=0.002	0.066 p<0.001	-0.038 p=0.009	0.118 p=0.008	0.011 *p* = 0.442	-0.007 *p* = 0.635
Margarine	0.073 *p* = 0.104	0.033 p=0.071	-0.054 p=0.001	0.070 p<0.001	-0.037 *p* = 0.309	-0.040 p=0.021
Olive oil or other vegetable oil/shortening or lard	0.127 p=0.001	0.020 *p* = 0.151	-0.033 p=0.008	0.062 p<0.001	0.014 *p* = 0.25	-0.101 p=0.001
I do not eat oils and fats at least 5 times per week	0.082 p=0.020	-0.011 *p* = 0.578	0.033 *p* = 0.117	0.064 p<0.001	0.024 *p* = 0.26	-0.016 *p* = 0.455
I eat a combination of these oils and fats at least 5 times per week	-0.001 *p* = 0.984	-0.002 *p* = 0.933	0.124 p<0.001	-0.029 *p* = 0.11	0.007 p=0.74	0.095 p<0.001
Missing	-	-	-	-	-	-

^1^indicates a very strong Kappa value

^2^indicates a substantial Kappa value

*indicates question was not mutually exclusive

Turquoise: indicates p<0.001

Bright Green: indicates p<0.05

Yellow: indicates p<0.1

In Table 4 parental responses were stratified by child’s level of ASD. The results did not show the expected agreements between some reported eating patterns (vegetables, whole grains) and increasing level of ASD. We also observed several inconsistencies, such as individuals in Level 4 reportedly having lack of diet variety overall, but we also found higher diet variety within specific food groups.

**Table 4:** Percent of reponses and parent-child agreement to the survey questions by ASD level (N = 488).

	ASD Level 1 Agreement	ASD Level 2 Agreement	ASD Level 3 Agreement		ASD Level 4 Agreement
**1. Do you eat a variety of food groups (different fruits, vegetables, meats, grains, fats and oils) in your daily diet?**					
Response	Kappa and *P*-value	Kappa and *P*-value		Kappa and *P*-value	Kappa and *P*-value
Yes	0.357 p<0.001	0.04 p<0.001		0.02 p=0.002	0.00 *p* = 0.981
No	0.006 *p* = 0.625	0.05 *p* = 0.156		-0.01 *p* = 0.82	0.06 p=0.002
Missing	-	-		-	-
**2. Do you eat the same foods every day?**					
Response	Kappa and *P*-value	Kappa and *P*-value		Kappa and *P*-value	Kappa and *P*-value
Yes	0.002 *p* = 0.962	0.07 p=0.001		-0.00 *p* = 0.918	0.05 p<0.001
No	0.117 p<0.001	0.05 *p* = 0.109		0.02 p=0.005	-0.01 *p* = 0.262
Missing	-	-		-	-
**3. Do you like to snack (eat small amounts between meals)?**					
Response	Kappa and *P*-value	Kappa and *P*-value		Kappa and *P*-value	Kappa a nd *P*-value
Yes	0.221 p<0.001	0.05 p=0.001		0.01 *p* = 0.615	-0.01 *p* = 0.418
No	0.054 p=0.001	-0.00 *p* = 0.981		0.05 p=0.003	0.02 *p* = 0.125
Sometimes	0.009 *p* = 0.42	0.04 p=0.018		-0.06 *p* = 0.141	0.04 p=0.037
Rarely	0.004 *p* = 0.271	-0.01 *p* = 0.291		0.02 *p* = 0.201	-0.01 *p* = 0.704
Missing	-	-		-	-
**4. If you snack, which type of snack food do you prefer most of the time?**					
Response	Kappa and *P*-value	Kappa and *P*-value		Kappa and *P*-value	Kappa and *P*-value
Sweet and Soft	0.224 p<0.001	-0.03 *p* = 0.115		0.04 p=0.018	-0.01 *p* = 0.577
Sweet and Crunchy	0.039 p=0.004	0.01 *p* = 0.852		0.01 *p* = 0.503	0.01 *p* = 0.702
Salty and Soft	0.001 *p* = 0.953	0.02 *p* = 0.132		0.03 *p* = 0.521	0.02 *p* = 0.248
Salty and Crunchy	0.040 p=0.008	0.06 p=0.001		-0.02 *p* = 0.225	-0.03 *p* = 0.366
Fruit or Vegetables	0.014 *p* = 0.181	0.023 *p* = 0.181		0.002 *p* = 0.915	-0.005 *p* = 0.818
No preference	-0.025 p=0.020	0.046 p=0.006		0.031 *p* = 0.144	0.093 p<0.001
Missing	-	-		-	-
**5. Do you drink at least 8 glasses of water every day?**					
Response	Kappa and *P*-value	Kappa and *P*-value		Kappa and *P*-value	Kappa and *P*-value
Yes	0.302 p<0.001	0.031 p=0.051		-0.015 *p* = 0.214	0.003 *p* = 0.712
No	0.027 p=0.087	0.092 p=0.027		0.060 p<0.001	0.016 *p* = 0.139
Missing	-	-		-	-
**6. Which types of beverages/drinks do you prefer?**					
Response	Kappa and *P*-value	Kappa and *P*-value		Kappa and *P*-value	Kappa and *P*-value
Hot	0.056 p=0.059	0.008 *p* = 0.663		0.051 p=0.012	-0.015 *p* = 0.387
Cold	0.056 p<0.001	0.098 p=0.016		0.012 *p* = 0.358	0.016 *p* = 0.102
Both	0.051 p=0.001	0.037 p=0.049		0.018 *p* = 0.584	0.012 *p* = 0.296
Neither	0.023 p=0.002	-0.010 *p* = 0.426		-0.022 *p* = 0.251	-0.032 *p* = 0.457
Missing	-	-		-	-
**7. Do you take nutrition supplements?**					
Response	Kappa and *P*-value	Kappa and *P*-value		Kappa and *P*-value	Kappa and *P*-value
Yes	0.238 p<0.001	0.051 p=0.001		0.006 *p* = 0.593	-0.006 *p* = 0.471
No	0.054 p=0.001	0.025 *p* = 0.56		0.035 p=0.018	0.029 p=0.008
Missing	-	-		-	-
**8. If you take supplements, which type do you take?**					
Response	Kappa and *P*-value	Kappa and *P*-value		Kappa and *P*-value	Kappa and *P*-value
Daily multivitamin and minerals combined	0.222 p<0.001	0.026 *p* = 0.134		0.009 *p* = 0.489	-0.003 *p* = 0.779
Single nutrient or minerals	0.029 p=0.032	0.092 p=0.026		-0.021 *p* = 0.298	-0.003 *p* = 0.858
Both	0.039 p=0.003	0.006 *p* = 0.772		0.018 *p* = 0.663	-0.015 *p* = 0.366
Missing	-	-		-	-
**9. Do you remember what type of eater you were when you were less than 5 years old?**					
Response	Kappa and *P*-value	Kappa and *P*-value		Kappa and *P*-value	Kappa and *P*-value
I do not remember	0.029 *p* = 0.264	0.035 p=0.047		0.006 *p* = 0.790	-0.009 *p* = 0.636
I ate everything my parents gave me	0.048 p=0.002	0.027 *p* = 0.541		0.005 *p* = 0.781	0.030 p=0.025
I was a picky eater and didn’t like certain foods	0.079 p<0.001	0.038 p=0.011		0.055 p=0.022	0.001 *p* = 0.914
Missing	-	-		-	-
**10. Do you now like some of the foods you didn’t like as a child?**					
Response	Kappa and *P*-value	Kappa and *P*-value		Kappa and *P*-value	Kappa and *P*-value
Yes	0.232 p<0.001	0.024 *p* = 0.133		0.025 p=0.033	0.004 *p* = 0.671
No	0.052 p=0.001	0.114 p=0.007		0.006 *p* = 0.672	0.016 *p* = 0.134
Missing	-	-		-	-
**11. If you eat different vegetables, which do you eat at least 5 times per week?** *					
Response	Kappa and *P*-value	Kappa and *P*-value		Kappa and *P*-value	Kappa and *P*-value
Dark leafy greens	0.118 p=0.001	-0.010 *p* = 0.601		0.012 *p* = 0.499	0.012 *p* = 0.423
Orange	0.059 p<0.001	0.088 p=0.031		0.013 *p* = 0.334	-0.007 *p* = 0.466
Cruciferous	0.064 p<0.001	0.023 *p* = 0.231		-0.061 p=0.091	-0.005 *p* = 0.716
White and starchy	0.044 p=0.004	0.073 p<0.001		-0.002 *p* = 0.893	-0.025 *p* = 0.202
Onion family	0.033 p=0.032	0.018 *p* = 0.359		0.044 p=0.013	-0.017 *p* = 0.212
I do not eat any vegetable groups at least 5 times per week	0.024 p=0.033	-0.009 *p* = 0.607		0.021 *p* = 0.338	-0.008 *p* = 0.672
I eat a combination of these vegetable groups at least 5 times per week	-0.003 *p* = 0.820	0.007 *p* = 0.710		0.041 p=0.041	0.066 p<0.001
Missing	-	-		-	-
**12. If you eat different meats, which do you eat at least 5 times per week?** *					
Response	Kappa and *P*-value	Kappa and *P*-value		Kappa and *P*-value	Kappa and *P*-value
Fish	0.126 p=0.001	0.004 *p* = 0.836		0.003 *p* = 0.847	-0.009 *p* = 0.505
Chicken or Turkey	0.076 p<0.001	0.040 *p* = 0.288		0.010 *p* = 0.384	-0.004 *p* = 0.656
Red meat	0.047 p<0.001	0.055 p<0.001		0.031 *p* = 0.191	-0.006 *p* = 0.424
Cold cuts and other ready-to-eat meats	0.031 p=0.042	0.044 p=0.025		-0.003 *p* = 0.847	-0.019 *p* = 0.505
I do not eat meats at least 5 times per week	-0.006 p=0.024	0.036 *p* = 0.024		-0.004 *p* = 0.858	0.023 *p* = 0.286
I eat a combination of these meats at least 5 times per week	-0.003 *p* = 0.838	0.009 *p* = 0.623		0.038 p=0.067	0.060 p=0.001
Missing	-	-		-	-
**13. If you eat different grains, which do you eat at least 5 times per week?** *					
Response	Kappa and *P*-value	Kappa and *P*-value		Kappa and *P*-value	Kappa and *P*-value
White bread	0.204 p<0.001	0.030 p=0.072		-0.011 *p* = 0.380	-0.003 *p* = 0.714
Whole grain breads	0.077 p<0.001	0.026 *p* = 0.534		-0.008 *p* = 0.576	-0.004 *p* = 0.664
Breakfast Cereals	0.052 p=0.001	0.061 p=0.001		-0.045 *p* = 0.137	-0.014 *p* = 0.170
Rice	-0.006 *p* = 0.664	0.050 p=0.012		0.019 *p* = 0.310	0.052 p=0.099
Pasta	0.028 p=0.074	0.026 *p* = 0.175		0.013 *p* = 0.447	0.012 *p* = 0.368
I do not eat grains at least 5 times per week	-0.008 *p* = 0.294	0.019 *p* = 0.160		0.030 *p* = 0.125	0.012 *p* = 0.575
I eat a combination of these grains at least 5 times per week	0.012 *p* = 0.397	-0.015 *p* = 0.459		0.061 p=0.002	0.045 p=0.003
Missing	-	-		-	-
**14. If you use different oils and fats, which do you eat at least 5 times per week?** *					
Response	Kappa and *P*-value	Kappa and *P*-value		Kappa and *P*-value	Kappa and *P*-value
Butter	0.124 p=0.005	0.048 p=0.007		0.001 *p* = 0.955	-0.005 *p* = 0.639
Margarine	0.048 p=0.002	0.115 p=0.010		-0.017 *p* = 0.282	-0.021 p=0.085
Olive oil or other vegetable oil/shortening or lard	0.077 p<0.001	0.032 p=0.045		0.010 *p* = 0.703	-0.015 p=0.075
I do not eat oils and fats at least 5 times per week	0.030 p=0.012	-0.008 *p* = 0.654		0.022 *p* = 0.296	-0.057 *p* = 0.151
I eat a combination of these oils and fats at least 5 times per week	-0.005 *p* = 0.743	0 *p* = 0.999		0.054 p=0.008	0.070 p<0.001
Missing	-	-		-	-

^1^indicates a very strong Kappa value

^2^indicates a substantial Kappa value

*indicates question was not mutually exclusive

Turquoise: indicates p<0.001

Bright Green: indicates p<0.05

Yellow: indicates p<0.1

## Discussion

This pilot study was conducted to explore the degree to which parents perceive their own dietary intake patterns to be similar with the intake patterns of their child with ADS. We selected 14 different questions to examine diet variety (2 questions), snacking (2 questions), beverage intake (2 questions), supplement intake (2 questions), picky eating (2 questions), and intake of food groups known to be problematic for children with ASD (10) (4 questions). We found that while perceived overall diet variety was expressed and had strong agreement, diet variety within food groups (reported consumption of food group variety across all food groups) was low (less than 11% of responses for “I eat a combination of…at least 5 times per week” for all questions).

Approximately 70% of young individuals with ASD have difficulties with dietary intake, including neophobia and feeding problems such as overeating and using food to self-soothe [17]. Since chronic overeating can lead to health complications, such as obesity and associated co-morbidities, developing interventions to help families and caretakers of children with ASD to learn healthy eating behaviors is of critical importance. One such possible point of intervention is the implementation of nutrition education for parents to help improve the parent’s intake behavior, which might serve as role model for their children’s food intake. For example, the introduction of fortified foods into the diet of both the parent and child could be a possible solution for current lack of nutrients due to diets low in variety [11]. As previous research in a nationally representative dataset indicated, the intergenerational transfer of eating habits has been observed for mothers and fathers (or female/male caretakers of children) [18] in young children, so one could assume that parents of children with ASD can be instrumental in teaching their children to have higher diet variety and better diet quality.

Although less than 10% of the sample parents and children in this study were classified as obese, we found substantial agreement in the obesity status between the parent-child dyads, indicating that parental influences on dietary intake behaviors contribute to children’s weight status. However, it is well established that genetic factors can have a large role in children’s obesity status and to date, 127 obesity-related genes have been identified [22]. Nevertheless, individuals with ASD have been found to be at much larger risk for chronic disease compared to the general population and efforts to improve diet quality might help alleviate this situation.

An important indicator of reduced disease risk is high diet variety, which has also been linked to reduced all-cause mortality [15]. The data of this study indicated a strong relationship between parental perception of their own and their child’s diet variety, offering a potentially highly effective path to healthier diet patterns in children, if parents can be motivated to serve as positive role models. In our investigation of eating patterns and specific components of diet, we found moderate to strong agreement of food group consumption for most food groups, with the single exception of the meat. Also, parental reports of picky eating, snacking and beverage consumption were at least moderately associated with their own, which further indicates a potential to improve the diets of children with ASD.

Some limitations of this study need to be highlighted. First, all data analysis was based on parental self-report and was therefore potentially biased. We did not use collection dietary data or other means to validate the information provided by the parents. Also, we collected this cross-sectional data over a relatively short time period and since food intake behaviors might change seasonally, our data might not be representative of typical or usual intake patterns over the course of a year. Other research focuses only on dietary components (dairy, for example) which is affected by ASD [23], while our study was designed to target the relationship between parental and parent’s perceived intake preferences of their child with ASD. Since this study was an exploratory pilot study, we did not have objectively measured values to compare parental responses to assess if the reported relationships were real or only perceived. Also, study participants received a small financial reward, which may have led to self-selection bias of individuals with higher financial need. On the other hand, one strength of this study is that we were able to recruit a large number of parents, which is unusual for this hard-to-reach population.

Surprisingly, we did not find some expected results, such as more reported “unhealthy” diet patterns with increasing BMI status or lack of diet variety and low intakes of problematic food groups, such as vegetables and whole grains, with increasing level of severity of ASD. Our results showed that parents perceive a high level of agreement between their own and the diet patterns they reported for their child with ASD, however, our data indicates the needs for future studies including larger and diverse samples, a more detailed survey, direct observation or other more objective measures of diet patterns to further pursue the potential for including parents as agents of change in efforts to improve dietary intake in children with ASD and lower the risk for chronic diseases in children with ASD. Also, many parents of children with ASD might not be aware of the pivotal role they have in their child’s dietary pattern development.

## Conclusion

Results from this exploratory study indicate perceived intergenerational transfer of some eating behaviors between parents and their adult children with ASD. Parental reports showed similarities in diet variety, not eating the same foods every day, orange, white and starchy vegetable intake, and eating a combination of different vegetables every week, but not in meat intake. This new knowledge can be used to develop more detailed and in-depth tools to better understand the role of parents on the diets of their children with ASD to ultimately design interventions which support efforts to prevent chronic diseases in individuals with ASD throughout the life course.

## Supporting information

S1 Data (XLSX)
